# Age-Aware Utility Maximization in Relay-Assisted Wireless Powered Communication Networks

**DOI:** 10.3390/e23091177

**Published:** 2021-09-07

**Authors:** Ning Luan, Ke Xiong, Zhifei Zhang, Haina Zheng, Yu Zhang, Pingyi Fan, Gang Qu

**Affiliations:** 1 School of Computer and Information Technology, Beijing Jiaotong University, Beijing 100044, China; 19120388@bjtu.edu.cn (N.L.); 18112041@bjtu.edu.cn (H.Z.); 2 Beijing Key Laboratory of Traffic Data Analysis and Mining, Beijing Jiaotong University, Beijing 100044, China; 3 Frontiers Science Center for Smart High-Speed Railway System, Beijing Jiaotong University, Beijing 100044, China; 4 State Grid Energy Research Institute Co., Ltd., Beijing 102209, China; zhangyu2@sgeri.sgcc.com.cn; 5 Department of Electronic Engineering, Tsinghua University, Beijing 100084, China; fpy@tsinghua.edu.cn; 6 Beijing National Research Center for Information Science and Technology, Tsinghua University, Beijing 100084, China; 7 Department of Electrical and Computer Engineering, University of Maryland at College Park, College Park, MD 20742, USA; gangqu@umd.edu

**Keywords:** wireless powered communication networks, real-time state update, age of information, utility maximization

## Abstract

This article investigates a relay-assisted wireless powered communication network (WPCN), where the access point (AP) inspires the auxiliary nodes to participate together in charging the sensor, and then the sensor uses its harvested energy to send status update packets to the AP. An incentive mechanism is designed to overcome the selfishness of the auxiliary node. In order to further improve the system performance, we establish a Stackelberg game to model the efficient cooperation between the AP–sensor pair and auxiliary node. Specifically, we formulate two utility functions for the AP–sensor pair and the auxiliary node, and then formulate two maximization problems respectively. As the former problem is non-convex, we transform it into a convex problem by introducing an extra slack variable, and then by using the Lagrangian method, we obtain the optimal solution with closed-form expressions. Numerical experiments show that the larger the transmit power of the AP, the smaller the age of information (AoI) of the AP–sensor pair and the less the influence of the location of the auxiliary node on AoI. In addition, when the distance between the AP and the sensor node exceeds a certain threshold, employing the relay can achieve better AoI performance than non-relaying systems.

## 1. Introduction

With the large-scale deployment of the Internet of things (IoT) devices in applications such as environment surveillance and industrial control [1,2], status update systems that report real-time system information become increasingly more important. In such systems, it is required to make accurate decisions based on fresh information update and measurement of information freshness becomes necessary. However, traditional network performance metrics like delay and throughput cannot characterize the information freshness. Therefore, the concept of age of information (AoI) was introduced as the time duration from the generation time of the latest received status update packet to the current time moment [3]. For real-time update systems, the goal is to get status update information as fresh as possible, which can be considered as the minimization of AoI.

In IoT systems, information is normally collected by the edge devices or sensors. These sensors are generally powered by batteries of limited capacity, which require to be replaced or recharged periodically. However, battery replacement or frequent recharging is labor-intensive and could be impossible, in particular in large-scale network scenarios and harsh environments. For this, energy harvesting (EH) technology has emerged as a promising alternative to collect energy from the external environment to power the low-power IoT devices. It is believed that EH has great potential in the future sixth-generation (6G) communication networks. Therefore, it is also expected to be used in future industrial control, environment monitoring, and other real-time IoT applications.

Existing EH technologies are of two types: traditional natural energy source and radio-frequency (RF) signal-based energy source. Compared with traditional natural energy sources [4,5], RF signals are easy to control, can provide steady power, and have relatively low requirements on the deployment environment [6]. Evidently, we have seen many works on AoI-based wireless powered communication networks (WPCN) powered by RF EH [7,8,9,10,11,12,13]. In [7], an optimal online state update strategy to minimize the AoI over long-term time scale with energy constraints was studied. In [8], the performance of AoI in WPCN was analyzed, and it proved that the smaller the probability of packet generation, the smaller the average AoI. In [9], the emergency AoI (U-AoI) in WPCN was minimized. In [10], the uplink AoI in two-way wireless powered networks was discussed, where the nonlinear AoI expression was adopted. In [11], the AoI performance limit for the actual wireless power transfer (WPT) network was explored. In [12], the trade-off between storable energy and system AoI in the WPT network was investigated, and in [13], the optimal design of AoI-based fog computing WPCN was studied.

The above works only considered the basic three-node network model. Recently, researchers have extended this to the study of AoI performance in multiple-node networks. For example, the authors of [14] minimized the AoI in large-scale WPCN with multi-sensor nodes, and derived the solution of the average charging time of nodes in the network. The authors of [15] studied the WPT-powered networks and explored when to terminate energy collection and how to properly assign resources for uploading data in order to minimize AoI. In [16], an optimal online sampling strategy for joint optimization of update packet transmit was presented to minimize the AoI, and a deep reinforcement learning (DRL) approach was proposed to effectively learn the optimal AoI strategy. In [17], the AoI in WPCN with multi-user scheduling based on non-orthogonal multiple-access (NOMA) was discussed, and the closed-form expression of AoI was derived. However, the above work only considered the single-hop network. Due to factors such as too far or obstructions between the sensor and the AP, the connection between them cannot always be established. Therefore, relaying technology can be employed to help sensors transmit information to their sink node [18,19,20,21]. Particularly, in [18], a cooperative WPCN with flat fading was studied, in which a source and a relay collected energy from a remote power station. In [19], the AoI in a cooperative wireless communication system with synchronous wireless information and power transmission (SWIPT) was studied, where two protocols were discussed.

We observe that in most of these works on AoI-based relay-assisted WPCNs, it was assumed that the relay node (or auxiliary node) could directly participate in charging or information transmission. As a matter of fact, this may not be realistic in real-life WPCN because relay nodes may also have limited energy and thus are reluctant or refuse to collaborate in the transmission of energy and/or information. We consider this selfish nature of the relay nodes in this article and propose to design an effective incentive mechanism to improve the system AoI performance. In our proposed mechanism, the access point (AP) will coordinate auxiliary nodes to charge the sensor node based on RF until a sufficient amount of energy is harvested by the sensor node; then, the sensor node will send real-time status update information to the AP. Unlike a similar work [22] that uses a vague incentive mechanism and assumes the sensor node transmit packets directly to the AP, we use the spectrum priority as the incentive for the auxiliary nodes to participate in the charging process, and use the auxiliary node as the relay on the rout of the status update packets from the sensor node to the AP. For such a system, utility functions are defined based on AoI for AP and auxiliary nodes respectively, and the utility maximization problems are formulated. In order to achieve a win–win benefit, we apply the Stackelberg game model to design the effective cooperation between the AP-sensor pair and auxiliary node.

We make the following key contributions in this article.

First, we extend the previous work on WPCN to the more realistic scenarios of relay-assisted WPCN with selfish auxiliary nodes. We describe the system model and propose a protocol to encourage the cooperation between AP and auxiliary node to keep information fresh.

Second, we introduce utility functions for the AP-sensor pair and the auxiliary node and then establish a Stackelberg game in order to improve the system’s performance. To solve the non-convex utility maximization problem, we use the Lagrangian method based on a new slack variable and are able to obtain the optimal solution in the closed form.

Third, we conduct numerical simulations to show that larger transmit power of the AP results in smaller AoI and less dependency on the location of the auxiliary nodes. In addition, when the distance between the AP and the sensor exceeds a certain threshold, employing the relay can achieve better AoI performance than the current non-relaying systems.

The rest of this article is organized as follows. We elaborate the system model of relay-assisted WPCN in the next section and then formulate the AoI-aware utility maximization problem in Section 3. Our proposed solution to this problem is explained in detail in Section 4. The simulation results are reported in Section 5 before we conclude the article.

## 2. System Model

**Figure 1:**
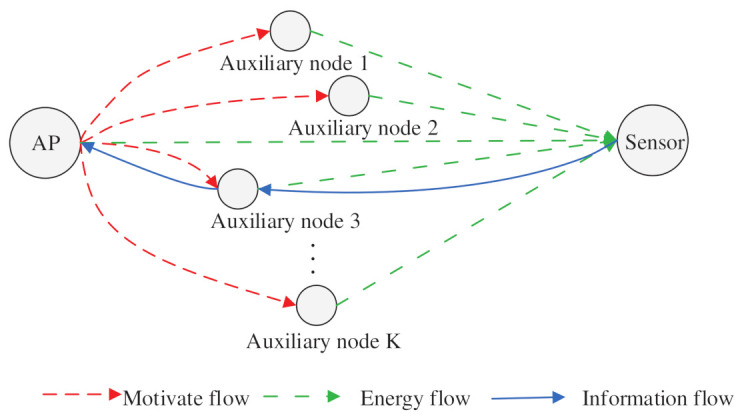
Illustration of the relay-assisted WPCN model.

Consider a WPT-driven network, as illustrated in Figure 1, which includes an AP, multiple auxiliary nodes, and a sensor node with limited energy. In this system, the AP needs to collect status update information from the sensor and this is performed in two stages: first, the AP broadcasts the accessible bandwidth resources to the auxiliary node as an incentive to participate in the cooperation to charge the sensor, and the auxiliary node judges whether to participate in the power supply to the sensor according to the received incentive. When the sensor harvests enough energy, it utilizes the accumulated energy to send the status update to the AP. In the network, the frequency band authorized by the AP is fixed, so multiple auxiliary nodes compete to act as the helping node and only one auxiliary node will be selected to help charge the sensor node. The selected auxiliary node is allowed to use the bandwidth resource and can be used as a relay.

We use *k* to denote the *k*-th auxiliary node, where k∈{1,…,K}. We use *i* to denote the index of the data package, where i∈{1,2,…,I}. We assume that the AP has steady power supply and the energy and information is transmitted through orthogonal channels to avoid any interference. We further assume that all wireless links expose in additive white Gaussian noise.

### 2.1. Energy Harvesting Model

Let $P_{AP}$ and $P_{k}$ denote the transmit power of AP and the *k*-th auxiliary node, respectively. Let $h_{a,d}\left(t\right)$ and $h_{k,d}\left(t\right)$ be the wireless channel gain between the AP and the sensor and between the *k*-th auxiliary node and the sensor at time *t*, respectively. Let $b^{\left[i\right]}$ be the time when the *i*-th packet starts transmission. We assume that the sensor’s battery capacity is $B_{s}$ Joule. Once the battery is fully charged, the sensor node is triggered to send a newly generated packet with the harvested energy. It is well known that energy is the integral of power over time. Considering the energy harvesting efficiency and our system model, the accumulated energy by RF-based EH for data packet *i* can be expressed as

(1) $$E_{h}^{\left[i\right]}=\int_{b^{\left[i-1\right]}}^{b^{\left[i\right]}}\eta \left(P_{AP}|h_{a,d}\left(t\right){|}^{2}+P_{k}|h_{k,d}\left(t\right){|}^{2}\right)dt,$$

where η∈(0,1) denotes the energy harvesting efficiency. Note that $b^{\left[i\right]}$ is the time when the battery is fully charged, therefore, we have

(2) $$B_{s}=E_{h}^{\left[i\right]}.$$

We partition the time into *I* subintervals at time instants, i.e., 0 = $b^{\left[0\right]}$ < $b^{\left[1\right]}$ < ... < $b^{\left[i-1\right]}$ < $b^{\left[i\right]}$ < ... < $b^{\left[I\right]}$, where the *i*-th subinterval [$b^{\left[i-1\right]}$, $b^{\left[i\right]}$] is the time that the sensor harvests energy for information packet *i*. The length of this subinterval is

(3) $$T_{s}^{\left[i\right]}=b^{\left[i\right]}-b^{\left[i-1\right]}.$$

Let $d^{\left[i\right]}$ denote the arriving time of the *i*-th information packet at the AP, i.e., the time when the packet *i* transmission is completed. Thus, the transmitting time of the *i*-th information packet is expressed by

(4) $$T_{t}^{\left[i\right]}=d^{\left[i\right]}-b^{\left[i\right]}.$$

Once the *i*-th information packet arrives at the AP, the sensor node can start generating the (i+1)-th packet if the status information becomes available. Therefore, we have the following:

(5) $$T_{t}^{\left[i\right]}\leq T_{s}^{\left[i+1\right]}.$$

The auxiliary relay node has a single antenna and adopts the decoding and forwarding relaying strategy. That is, decoding the received information before encoding and forwarding. The relay node is installed with an information decoder, an encoder, and energy storage. The length of the information packet is denoted by *L*.

Let $T_{t1}^{\left[i\right]}$ and $T_{t2}^{\left[i\right]}$ be the transmitting time of the *i*-th packet from the sensor to the *k*-th auxiliary node and from the *k*-th auxiliary node to the AP, respectively. According to Shannon theory, the rate can be calculated in terms of the signal-to-noise ratio (SNR) and bandwidth, so we have the expression of transmitting time, i.e.,

(6) $$T_{t1}^{\left[i\right]}=\frac{L}{W\log\left(1+\gamma_{1}\right)}\;\mathrm{and}\;T_{t2}^{\left[i\right]}=\frac{L}{W\log\left(1+\gamma_{2}\right)},$$

where *W* represents the bandwidth, $\gamma_{1}$ and $\gamma_{2}$ are the received SNR of the two communication links, that between sensor and the *k*-th auxiliary node and that between the *k*-th auxiliary node and the AP, respectively.

Considering the situation that the AP can collect the update packet directly from the sensor node, we can see that the total transmitting time of the *i*-th update packet from the sensor to the AP will be

(7) $$T_{t}^{\left[i\right]}=\min\left(\frac{L}{W\log\left(1+\gamma_{1}\right)}+\frac{L}{W\log\left(1+\gamma_{2}\right)},\frac{L}{W\log\left(1+\gamma \right)}\right).$$

Finally, the SNRs are given by

(8) $$\gamma =\frac{P_{d}|h_{d,a}\left(t\right){|}^{2}}{N_{0}W},\gamma_{1}=\frac{P_{d}|h_{d,k}\left(t\right){|}^{2}}{N_{0}W}\;\mathrm{and}\;\gamma_{2}=\frac{P_{k}^{\prime }|h_{k,a}\left(t\right){|}^{2}}{N_{0}W},$$

where $|h_{d,a}\left(t\right){|}^{2}$, $|h_{d,k}\left(t\right){|}^{2}$ and $|h_{k,a}\left(t\right){|}^{2}$ denote the wireless channel power gain between the sensor and the AP, between the sensor and the auxiliary node *k*, and between the auxiliary node *k* and the AP, respectively. $N_{0}$ represents the noise spectral density. $P_{d}$ and $P_{k}^{\prime }$ are the transmit power of the sensor and the *k*-th auxiliary node, respectively.

### 2.2. AoI Modeling

The current information packet’s AoI, i.e., packet *i* at time *t*, is described by

(9) $$\Delta^{\left[i\right]}\left(t\right)=t-U^{\left[i\right]}\left(t\right),$$

where $U^{\left[i\right]}$ (t) represents the time of generating the latest update packet received by AP, i.e.,

(10) $$U^{\left[i\right]}\left(t\right)=\max\left\{b^{\left[j\right]}\left|d^{\left[j\right]}\leq t\right.\right\}.$$

**Figure 2:**
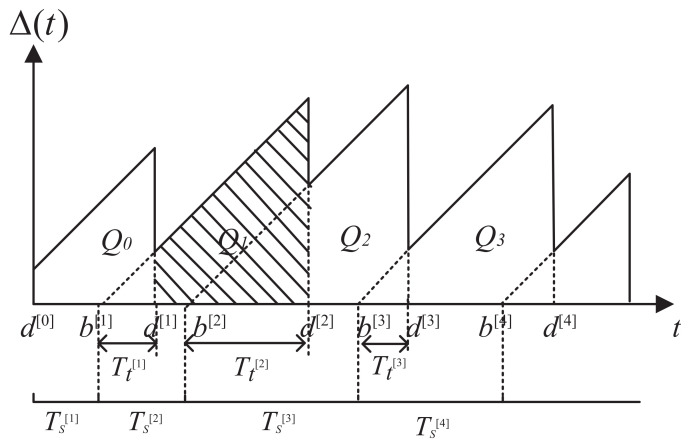
Evolution of the AoI.

Figure 2 illustrates the evolution AoI versus time. It is seen from the figure that when the destination node does not receive the state update packet, AoI increases linearly with time, which shows a sawtooth shape. When the destination node receives a new data packet, the AoI is reset to the delay that the status update experiences. In the time interval $\left[d^{\left[i\right]},d^{\left[i+1\right]}\right]$, the integral of AoI is the area under the $\Delta^{\left[i\right]}\left(t\right)$ curve. Therefore, its average AoI is expressed by

(11) $${\bar{\Delta }}^{\left[i\right]}=\frac{1}{d^{\left[i+1\right]}-d^{\left[i\right]}}\int_{d^{\left[i\right]}}^{d^{\left[i+1\right]}}\Delta^{\left[i\right]}\left(t\right)dt.$$

The integral term in the above formula is obtained by summing the area of $Q_{\left[i\right]}$,

(12) $$\int_{d^{\left[i\right]}}^{d^{\left[i+1\right]}}\Delta^{\left[i\right]}\left(t\right)dt=Q_{\left[i\right]},$$

which is expressed by

(13) $$Q_{\left[i\right]}=\frac{\left(T_{t}^{\left[i\right]}+T_{s}^{\left[i+1\right]}+T_{t}^{\left[i+1\right]}\right)\left(T_{s}^{\left[i+1\right]}-T_{t}^{\left[i\right]}+T_{t}^{\left[i+1\right]}\right)}{2}.$$

Therefore, the average AoI of the *i*-th data packet is expressed by

(14) $${\bar{\Delta }}^{\left[i\right]}=\frac{Q_{\left[i\right]}}{d^{\left[i+1\right]}-d^{\left[i\right]}}=\frac{1}{2}\left(T_{t}^{\left[i\right]}+T_{s}^{\left[i+1\right]}+T_{t}^{\left[i+1\right]}\right).$$

The harvesting energy time and transmitting update packet time are independent, so they could be regarded as independent and identically distributed variables. Therefore, for the long-term running, the system will be in a quasi-stationary state, which guarantees $T_{s}^{\left[i+1\right]}=T_{s}^{\left[i\right]}$ and $T_{t}^{\left[i+1\right]}=T_{t}^{\left[i\right]}$. In consequence, the average AoI is given by

(15) $$\bar{\Delta }=T_{t}+\frac{1}{2}T_{s}.$$

## 3. Problem Formulation

In the system described above, the auxiliary node may become selfish and not willing to charge the sensor due to their own limited energy. In order to motivate them to participate in charging the sensor node, the AP may use spectrum priority access as the incentive to encourage the auxiliary nodes. In this section, we will define the utility functions for the AP and the auxiliary node. We design a Stackelberg game method to build an effective cooperation between auxiliary node and AP–sensor pair. The basic idea of Stackelberg game is that one side is the leader and the other side is the follower. The leader acts first and the follower chooses his own action according to the leader’s strategy [23]. Their goal is to maximize their own interests.

**Figure 3:**
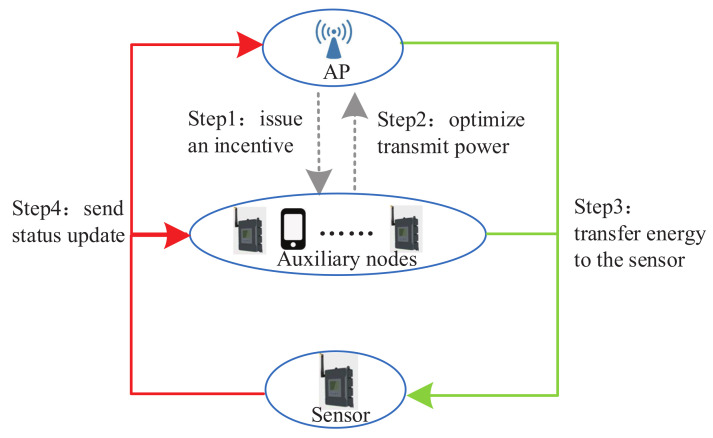
Overview of the incentive-based update packet collection system.

Specifically, first, the AP issues a certain bandwidth as the incentive for the auxiliary nodes to participate in charging. Second, the auxiliary nodes optimize their transmit power based on the incentive. Third, the AP optimizes its transmit power and then transfers energy to the sensor together with the selected auxiliary node. At last, the sensor utilizes the harvested energy to send update packets to the AP. The flow chart of the system is illustrated in Figure 3. Energy flow is shown in green on the right; update packet transmission flow is shown in red on the left.

### 3.1. Utilities of the Auxiliary Node

Let $\Gamma_{k}\left(x\right)$ denote the cost of the *k*-th auxiliary node to transmit energy to the sensor and information to the AP at power level *x*. Similar to the work in [24], we can model this cost as

(16) $$\Gamma_{k}\left(x\right)=a_{k}x^{2}-b_{k}x+c_{k},$$

where $a_{k}$, $b_{k}$, and $c_{k}$ are predetermined parameters related to auxiliary node *k*. If node *k* uses power level $P_{k}$ and $P_{k}^{\prime }$ to transmit to the sensor and to the AP, respectively, the utility of the auxiliary node *k* can be expressed by

(17) $$U_{k}\left(P_{k},P_{k}^{\prime },T_{s},T_{s}^{\prime }\right)=\alpha B-T_{s}\Gamma_{k}\left(P_{k}\right)-T_{s}^{\prime }\Gamma_{k}\left(P_{k}^{\prime }\right),$$

where αB is the incentive from the AP with α as the uniform factor of bandwidth and revenue, $T_{s}$ is the energy harvesting time and $T_{s}^{\prime }$ is the transmitting time from node *k* to the AP.

For the auxiliary node, it expects to maximize its utility. Therefore, the maximization problem $P_{k}$ is expressed by

(18) $$\begin{array}{c} P_{k}:\max_{P_{k},P_{k}^{\prime }}U_{k}\left(P_{k},P_{k}^{\prime },T_{s},T_{s}^{\prime }\right)\\ s.t.\;0<P_{k},P_{k}^{\prime }\leq P_{k}^{\max}, \end{array}$$

where $P_{k}^{\max}$ is the power threshold of the *k*-th auxiliary node.

### 3.2. Utilities of the AP

As mentioned earlier, the ratio factor of revenue to bandwidth is defined as α, and the overhead of AP is given by

(19) Ξ=αB.

Therefore, the utility associated with the sensor-AP pair is given by

(20) $$U_{AP}^{\left(0\right)}={\tilde{U}}_{AP}-\mu \bar{\Delta }-\omega T_{s}P_{AP}|h_{a,d}{|}^{2}-\Xi ,$$

where ${\tilde{U}}_{AP}$ is a constant, which is pre-defined. μ>0, ω>0 are the cost coefficient based on AoI and the cost coefficient based on energy, respectively.

Then, the AoI-based utility maximization problem is formulated as

(21) $$\begin{array}{c} P_{\mathrm{AP}}^{\left(1\right)}:\max_{P_{AP},T_{s}}U_{AP}^{\left(0\right)}\left(B,T_{s},P_{AP},P_{k}\right)\\ s.t.\;B_{s}\leq E_{h};T_{t}\leq T_{s};B\geq 0;0\leq P_{AP}\leq P_{AP}^{\max}, \end{array}$$

where $P_{AP}^{\max}$ is the maximum available power of the AP. As ${\tilde{U}}_{AP}$ is a constant, $P_{\mathrm{AP}}^{\left(1\right)}$ can be transformed to $P_{\mathrm{AP}}^{\left(2\right)}$, i.e.,

(22) $$\begin{array}{c} P_{\mathrm{AP}}^{\left(2\right)}:\min_{P_{AP},T_{s}}U_{AP}\left(B,T_{s},P_{AP},P_{k}\right)\\ s.t.\;B_{s}\leq E_{h};T_{t}\leq T_{s};B\geq 0;0\leq P_{AP}\leq P_{AP}^{\max}, \end{array}$$

where

(23) $$\begin{array}{c} U_{AP}\left(B,T_{s},P_{AP},P_{k}\right)=\mu \bar{\Delta }+\omega T_{s}P_{AP}|h_{a,d}{|}^{2}+\Xi \\ =\mu T_{t}+\frac{1}{2}\mu T_{s}+\omega T_{s}P_{AP}|h_{a,d}{|}^{2}+\alpha B. \end{array}$$

As μ, $T_{t}$, α, and *B* are fixed values, the problem $P_{\mathrm{AP}}^{\left(2\right)}$ is transformed to $P_{\mathrm{AP}}^{\left(3\right)}$, i.e.,

(24) $$\begin{array}{c} P_{\mathrm{AP}}^{\left(3\right)}:\min_{P_{AP},T_{s}}\frac{1}{2}\mu T_{s}+\omega T_{s}P_{AP}|h_{a,d}{|}^{2}\\ s.t.\;B_{s}\leq E_{h};T_{t}\leq T_{s};0\leq P_{AP}\leq P_{AP}^{\max}. \end{array}$$

## 4. Solution Method

We elaborate our proposed method and the derived solution for the above utility optimization problem.

### 4.1. Optimization of $P_{k}$ and $P_{k}^{\prime }$ with a Given $\left\{P_{AP},T_{s}\right\}$

As mentioned above, the problem $P_{k}$ is expressed by

(25) $$\begin{array}{c} P_{k}:\max_{P_{k},P_{k}^{\prime }}U_{k}\left(P_{k},P_{k}^{\prime },T_{s},T_{s}^{\prime }\right)\\ s.t.\;\;0<P_{k},P_{k}^{\prime }\leq P_{k}^{\max}, \end{array}$$

where

(26) $$U_{k}\left(P_{k},P_{k}^{\prime },T_{s},T_{s}^{\prime }\right)=\alpha B-T_{s}\Gamma_{k}\left(P_{k}\right)-T_{s}^{\prime }\Gamma_{k}\left(P_{k}^{\prime }\right).$$

By substituting the cost function expression into the above problem, problem $P_{k}$ can be expressed as follows,

(27) $$\begin{array}{c} P_{k}^{\left(1\right)}:\max_{P_{k},P_{k}^{\prime }}\left\{-a_{k}T_{s}{P_{k}}^{2}+b_{k}T_{s}P_{k}-a_{k}T_{s}^{\prime }{P_{k}^{\prime }}^{2}+b_{k}T_{s}^{\prime }P_{k}^{\prime }+\alpha B-c_{k}T_{s}-c_{k}T_{s}^{\prime }\right\}\\ s.t.\;\;0<P_{k},P_{k}^{\prime }\leq P_{k}^{\max}. \end{array}$$

**Lemma** **1.** *For a given $\left\{P_{AP},T_{s}\right\}$, the optimal solution to Problem $P_{k}$ is*

(28) $$\left\{\begin{array}{c} P_{k}^{*}=\frac{b_{k}}{2a_{k}},\\ P_{k}^{{}^{\prime }*}=\frac{b_{k}}{2a_{k}}. \end{array}\right.$$

**Proof.**  The objective function is about the quadratic function of two independent variables, and the second derivative is negative, so it is a concave function. The maximum value can be solved based on the quadratic formula directly. □

### 4.2. Optimization of $P_{AP}$ and $T_{s}$ with a Given $\left\{P_{k},P_{k}^{\prime }\right\}$

In problem $P_{\mathrm{AP}}^{\left(3\right)}$, two variables are multiple coupled, so the problem is non-convex. To tackle it, we introduce a new slack variable, i.e., $\pi =T_{s}\cdot P_{AP}$. As a result, the problem can be transformed into the following problem by variable substitution method:

(29) $$\begin{array}{c} P_{\mathrm{AP}}^{\left(4\right)}:\min_{T_{s},\pi }\frac{1}{2}\mu T_{s}+\omega |h_{a,d}{|}^{2}\pi \\ s.t.\;\;-T_{s}+T_{t}\leq 0\\ -T_{s}+\frac{1}{P_{AP}^{\max}}\pi \leq 0\\ -T_{s}-\frac{|h_{a,d}{|}^{2}}{P_{k}|h_{k,d}{|}^{2}}\pi +\frac{B_{s}}{\eta P_{k}|h_{k,d}{|}^{2}}\leq 0. \end{array}$$

As both of the objective function and the constraints of $P_{\mathrm{AP}}^{\left(4\right)}$ are linear, it is a convex problem, and the corresponding Lagrange function can be given by

(30) $$\begin{array}{c} L\left(T_{s},\pi ,\lambda \right)=\frac{1}{2}\mu T_{s}+\omega |h_{a,d}{|}^{2}\pi +\lambda_{1}\left(-T_{s}+T_{t}\right)+\lambda_{2}\left(-T_{s}+\frac{1}{P_{AP}^{\max}}\pi \right)\\ +\lambda_{3}\left(-T_{s}-\frac{|h_{a,d}{|}^{2}}{P_{k}|h_{k,d}{|}^{2}}\pi +\frac{B_{s}}{\eta P_{k}|h_{k,d}{|}^{2}}\right). \end{array}$$

The Karush–Kuhn–Tucker (KKT) condition of the problem is

(31a) $$\frac{1}{2}\mu -\lambda_{1}-\lambda_{2}-\lambda_{3}=0,$$

(31b) $$\omega |h_{a,d}{|}^{2}+\frac{1}{P_{AP}^{\max}}\lambda_{2}-\frac{|h_{a,d}{|}^{2}}{P_{k}|h_{k,d}{|}^{2}}\lambda_{3}=0,$$

(31c) $$\lambda_{1}\left(-T_{s}+T_{t}\right)=0,$$

(31d) $$\lambda_{2}\left(-T_{s}+\frac{1}{P_{AP}^{\max}}\pi \right)=0,$$

(31e) $$\lambda_{3}\left(-T_{s}-\frac{|h_{a,d}{|}^{2}}{P_{k}|h_{k,d}{|}^{2}}\pi +\frac{B_{s}}{\eta P_{k}|h_{k,d}{|}^{2}}\right)=0,$$

(31f) $$\lambda_{1}\geq 0,$$

(31g) $$\lambda_{2}\geq 0,$$

(31h) $$\lambda_{3}\geq 0,$$

(31i) $$-T_{s}+T_{t}\leq 0,$$

(31j) $$-T_{s}+\frac{1}{P_{AP}^{\max}}\pi \leq 0,$$

(31k) $$-T_{s}-\frac{|h_{a,d}{|}^{2}}{P_{k}|h_{k,d}{|}^{2}}\pi +\frac{B_{s}}{\eta P_{k}|h_{k,d}{|}^{2}}\leq 0.$$

According to (31b), the sum of the first two terms must be greater than 0, so $\lambda_{3}\neq 0$. Otherwise, (31b) will not hold.

It can be seen from (31a) and (31b) that $\lambda_{1}$ and $\lambda_{2}$ must not be 0 at the same time. Otherwise, $\lambda_{3}$ will be equal to two different values.

From (31c) and (31d), one can see that $\lambda_{1}$ and $\lambda_{2}$ cannot not be equal 0 at the same time. Otherwise, $T_{s}$ will be equal to two different values.

With above observations, there are two cases to analyze the solutions to the optimization problem.

Case 1: $\lambda_{1}=0$, $\lambda_{2}\neq 0$ and $\lambda_{3}\neq 0$, the optimal solution to $P_{\mathrm{AP}}^{\left(4\right)}$ is

(32) $$\left\{\begin{array}{c} T_{s}^{*}=\frac{B_{s}}{\eta \left(P_{k}|h_{k,d}{|}^{2}+P_{AP}^{\max}|h_{a,d}{|}^{2}\right)},\\ \pi^{*}=\frac{B_{s}P_{AP}^{\max}}{\eta \left(P_{k}|h_{k,d}{|}^{2}+P_{AP}^{\max}|h_{a,d}{|}^{2}\right)},\\ P_{AP}^{*}=P_{AP}^{\max}. \end{array}\right.$$

Case 2: $\lambda_{1}\neq 0$, $\lambda_{2}=0$ and $\lambda_{3}\neq 0$, the optimal solution to $P_{\mathrm{AP}}^{\left(4\right)}$ is

(33) $$\left\{\begin{array}{c} T_{s}^{*}=T_{t},\\ \pi^{*}=\left(\frac{B_{s}}{\eta P_{k}|h_{k,d}{|}^{2}}-T_{t}\right)\frac{P_{k}|h_{k,d}{|}^{2}}{|h_{a,d}{|}^{2}},\\ P_{AP}^{*}=\frac{\pi^{*}}{T_{t}}. \end{array}\right.$$

For the solutions of the two cases, it can be seen from the restrictive conditions that when (31i) is true, the solution is the case 1. Otherwise, it is the case 2. That is to say, the following formula is the judgment condition.

(34) $$\frac{B_{s}}{\eta \left(P_{k}|h_{k,d}{|}^{2}+P_{AP}^{\max}|h_{a,d}{|}^{2}\right)}\geq T_{t},$$

where the expression of $T_{t}$ is given by (7).

**Theorem** **1.** *The optimal solution to $P_{\mathrm{AP}}^{\left(4\right)}$ is expressed by*

(35) $$\left\{\begin{array}{c} T_{s}^{*}=\left\{\begin{array}{c} \frac{B_{s}}{\eta \left(P_{k}|h_{k,d}{|}^{2}+P_{AP}^{\max}|h_{a,d}{|}^{2}\right)},\;\;\;\;\;\;if\frac{B_{s}}{\eta \left(P_{k}|h_{k,d}{|}^{2}+P_{AP}^{\max}|h_{a,d}{|}^{2}\right)}\geq T_{t}\\ T_{t},\;\;\;\;\;\;\;\;\;\;\;\;\;\;\;\;\;\;\;\;\;\;\;\;\;\;\;\;\;\;\;\;\;\;otherwise \end{array}\right.\\ \pi^{*}=\left\{\begin{array}{c} \frac{B_{s}P_{AP}^{\max}}{\eta \left(P_{k}|h_{k,d}{|}^{2}+P_{AP}^{\max}|h_{a,d}{|}^{2}\right)},\;\;\;\;\;\;if\frac{B_{s}}{\eta \left(P_{k}|h_{k,d}{|}^{2}+P_{AP}^{\max}|h_{a,d}{|}^{2}\right)}\geq T_{t}\\ \left(\frac{B_{s}}{\eta P_{k}|h_{k,d}{|}^{2}}-T_{t}\right)\frac{P_{k}|h_{k,d}{|}^{2}}{|h_{a,d}{|}^{2}},\;\;\;otherwise \end{array}\right.\\ P_{AP}^{*}=\left\{\begin{array}{c} P_{AP}^{\max},\;\;\;\;\;\;\;\;\;\;\;\;\;\;\;\;\;\;\;\;\;\;\;if\frac{B_{s}}{\eta \left(P_{k}|h_{k,d}{|}^{2}+P_{AP}^{\max}|h_{a,d}{|}^{2}\right)}\geq T_{t}\\ \frac{\pi^{*}}{T_{t}}.\;\;\;\;\;\;\;\;\;\;\;\;\;\;\;\;\;\;\;\;\;\;\;\;\;\;\;\;otherwise \end{array}\right. \end{array}\right.$$

**Proof.**  As problem $P_{\mathrm{AP}}^{\left(4\right)}$ is convex, Theorem 1 can be proved with KKT conditions. □

## 5. Simulation Results

Some numerical results are shown to discuss the system performance in terms of achievable AoI, where the simulational parameters are set as follows. For clarity, the distance between the AP and the sensor is set to 6 m. The auxiliary nodes are randomly placed between AP and sensor. Assume that the distance between all auxiliary nodes and sensors is the same, that is, 3 m. Note that the location of the auxiliary nodes will be varied according to the purpose of the simulation. The channel is generated according to Rayleigh distribution. The path loss factor is set to 2. Other simulational parameters are summarized in Table 1.

**Figure 4:**
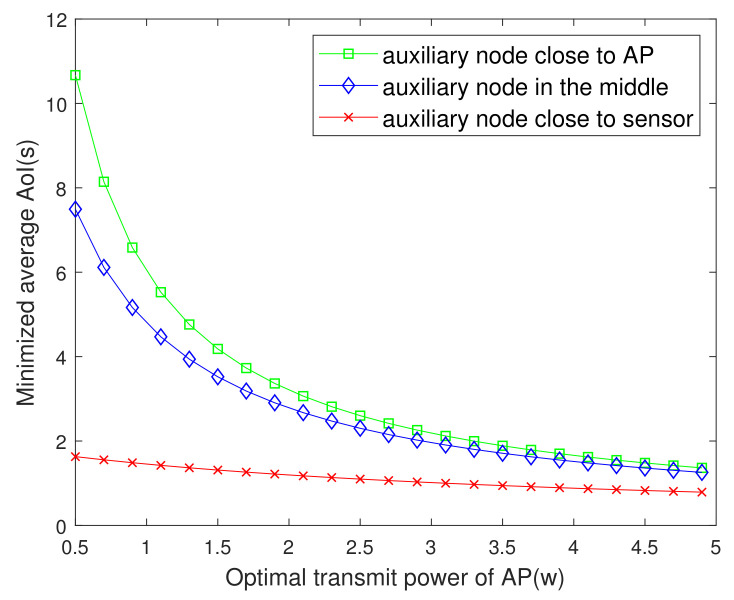
The minimized AoI versus $P_{AP}$.

Figure 4 plots the minimized average AoI versus the $P_{AP}$. One can observe that the AoI decreases gradually with the increase of $P_{AP}$. The reason is that increasing the AP’s transmit power will shorten the time for the energy harvested by the sensor to reach the battery capacity. Thus, it improves the freshness of the information. However, beyond a certain range, the change of AoI with transmit power is no longer significant because the average AoI is dominated by the information packet transmission time. This figure also shows the influence of different locations of auxiliary nodes on AoI is also different. The closer the auxiliary node to the sensor, the shorter time it will take to charge the sensor and the smaller the AoI.

**Figure 5:**
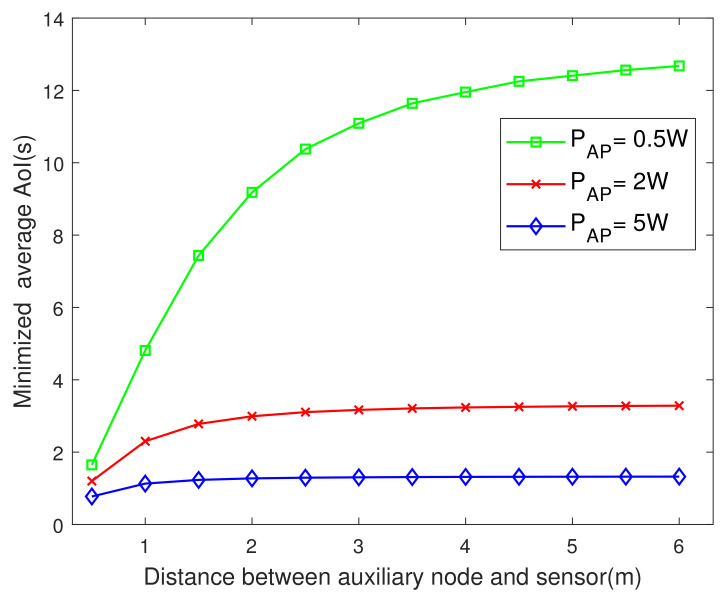
The minimized AoI versus $D_{kd}$.

Figure 5 plots the minimized AoI versus the distance between the auxiliary nodes and the sensor. It is seen that the closer the auxiliary node deployed to the sensor, the lower the AoI. This observation is consistent with the real situation as the closer the auxiliary node transmitting energy is to the sensor, the smaller the attenuation of electromagnetic wave is. Therefore, the time for sensor to collect energy will be reduced, so as to improve AoI. In addition, it is seen that the influence of the location on the AoI is different under different $P_{AP}$. The smaller the $P_{AP}$, the more obvious the influence of the location of the auxiliary node on AoI. When the $P_{AP}$ is small, the charging mainly depends on the auxiliary node, and the position of the auxiliary node is more critical. When the $P_{AP}$ is normal or larger, the $P_{AP}$ is the main factor affecting the AoI, so that the position of the auxiliary node has no obvious effect on AoI.

**Figure 6:**
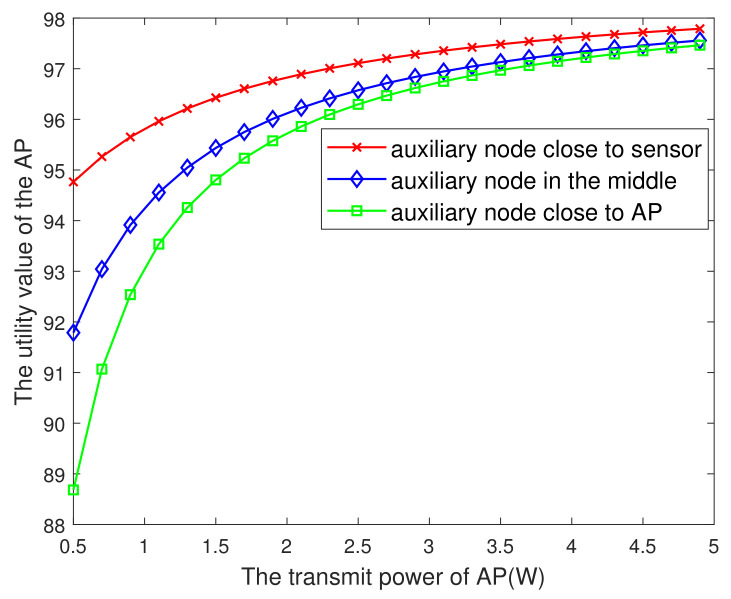
The AP’s utility value versus the $P_{AP}$.

Figure 6 plots the AP’s maximum utility value versus the transmit power of AP. From this figure, it is seen that the larger the $P_{AP}$, the greater the utility value, and finally tends to remain unchanged. The reason may be that in a certain range, by increasing the $P_{AP}$, although the cost of transmitting energy increases, it makes the information fresher. The benefit of AoI is greater than the cost of transmitting energy. Therefore, the utility value of the AP based on AoI becomes larger, and beyond a certain range, with the increase of $P_{AP}$, the impact on AoI is negligible and the increase of utility value is not obvious. Besides, it is also seen that the auxiliary nodes in different positions also have an impact on the utility value. The specific relationship is shown in the figure below.

**Figure 7:**
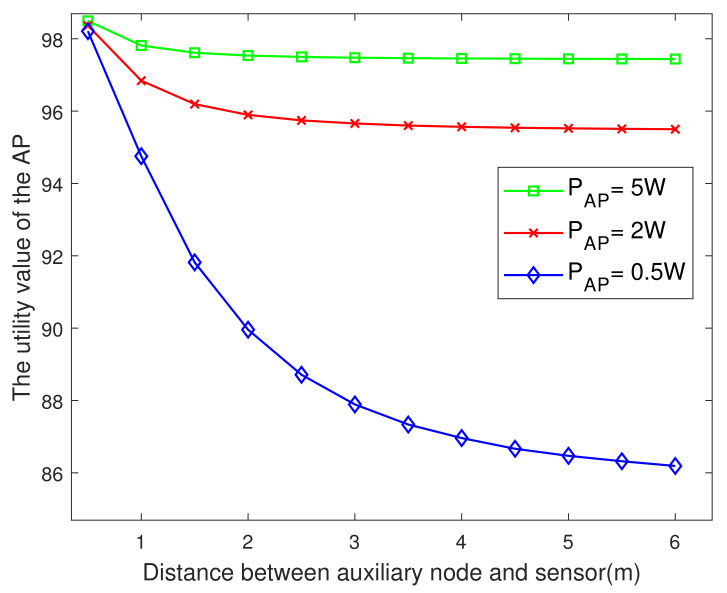
The AP’s utility value versus the $D_{ak}$.

Figure 7 plots the utility of AP versus the distance between the auxiliary nodes and the sensor. The shorter the distance between the auxiliary node and the sensor, the greater the AP’s utility value, and the smaller the AP transmit power, the more obvious that effect. The reason is similar to that associated with Figure 2.

**Figure 8:**
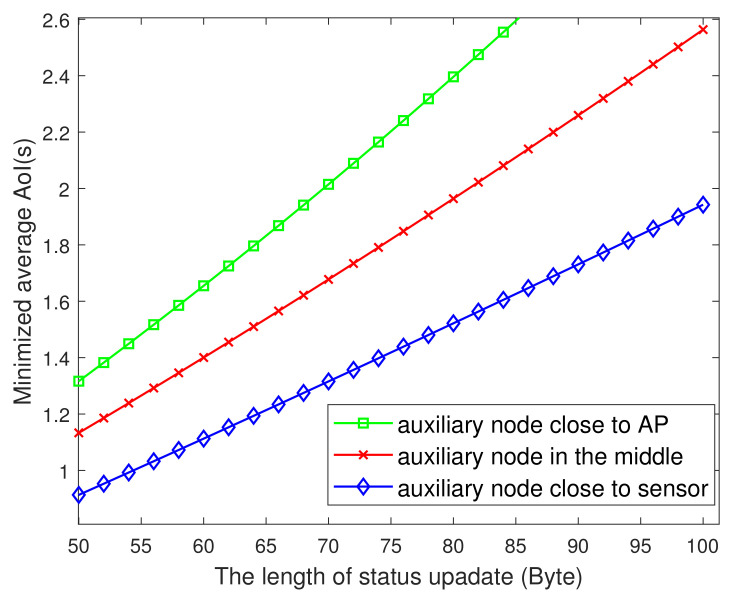
The minimized AoI versus L.

Figure 8 plots the change of AoI versus packet length. It is seen that the larger the length of the information packet, the larger the average AoI of the system. This may be because the length of the packet directly affects the transmit time of the packet. The larger the packet, the longer the transmitting time, which affects the freshness of the information. In addition, the influence of packet length on AoI varies with the location of auxiliary nodes. The closer the auxiliary node to the sensor, the less influence of packet length on AoI, because in the process of the auxiliary node serving as the relay and the sensor sending packets to the AP, the transmit power of the auxiliary node is larger than that of the sensor node, and the same packet length change has less impact on the transmit time.

**Figure 9:**
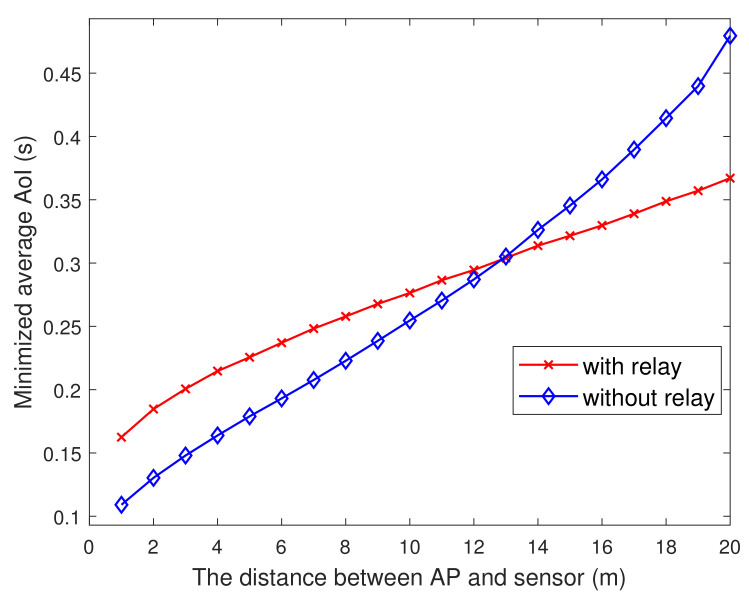
The minimized AoI versus $D_{ad}$.

Figure 9 depicts the change of AoI with the distance between the AP and the sensor. Obviously, the farther the distance, the larger the AoI. The figure also compares the AoI in the system with and without auxiliary node as the relay. When the distance is greater than 13 m, the information with auxiliary node as the relay is fresher when the sensor transmits update packets to the AP. Thus, for the actual system, if the relay within 13 m can choose not to activate them, it has important guiding significance.

**Figure 10:**
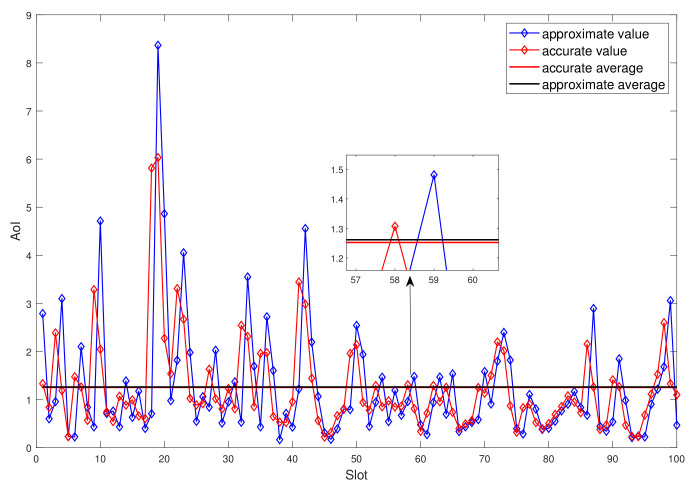
Accurate AoI versus approximate AoI.

Note that in this paper, the AoI is calculated by using the current packet’s transmit time to approximate the transmit time of the next packet, that is, using the current channel to approximate the channel of the next slot. Figure 10 shows the relationship between the approximate value and the exact value of AoI. It is seen that the change of the approximate value lags behind the exact value by one time slot. Due to the randomness of the channel, the approximate value may be larger or smaller than the true value. If the channel of the current time slot is better than the next time slot, the approximate value of the current AoI is a little smaller than the true value. If the channel of the current time slot is worse than the next time slot, the approximate value of the current AoI is larger than the real value. However, on average, the difference between our modeling method and the real value is very small, which indirectly proves the effectiveness of the modeling method.

## 6. Conclusions

In this article, we study a relay-assisted WPCN based on AoI with a focus on the case when the auxiliary nodes are selfish. The main idea behind our proposed solution is an incentive scheme that encourages the auxiliary nodes to collaborate. We formulate the problem and use the Stackelberg game theory to design an effective collaboration between AP–sensor pair and auxiliary node. More specifically, two utility functions for the AP–sensor pair and the auxiliary node were formulated. As maximizing the utility of the AP–sensor pair was non-convex, we transformed it into a convex problem by introducing a new slack variable, and then solved it by the Lagrangian method to obtain optimal solutions in the closed form. Simulation results showed that the larger the transmit power of the AP, the smaller the AoI and the less the influence of the location of the auxiliary node on AoI. In addition, when the distance from the AP to the sensor node exceeds a certain threshold, employing the relay can achieve better AoI performance than non-relaying systems. These results provide insightful and practical guidance for the design of relay-assisted WPCN in real life.

## Figures and Tables

**Table 1:** Parameter list.

Meaning	Parameter	Value
noise spectral density	$N_{0}$	$10^{-9}$ mW/Hz
bandwidth	*W*	1 kHz
maximum transmit power of k-th node	$P_{k}^{max}$	100 mW
maximum transmit power of sensor	$P_{d}^{max}$	10 mW
maximum transmit power of AP	$P_{AP}^{max}$	5 W
battery capacity	$B_{s}$	0.1 mj
energy harvesting efficiency	η	0.8
packet length	*L*	100 bytes
the cost coefficient based on AoI	μ	1
the cost coefficient based on energy	ω	$10^{4}$
the cost coefficient based on bandwidth	α	1
predetermined parameters	$a_{k}$	$1\times 10^{4}$
predetermined parameters	$b_{k}$	$2\times 10^{3}$
bandwidth incentive	*B*	100 Hz

## Data Availability

Not applicable.
